# OMT and tensor SVD–based deep learning model for segmentation and predicting genetic markers of glioma: A multicenter study

**DOI:** 10.1073/pnas.2500004122

**Published:** 2025-07-08

**Authors:** Zhengyang Zhu, Han Wang, Tiexiang Li, Tsung-Ming Huang, Huiquan Yang, Zhennan Tao, Zhong-Heng Tan, Jianan Zhou, Sixuan Chen, Meiping Ye, Zhiqiang Zhang, Feng Li, Dongming Liu, Maoxue Wang, Jiaming Lu, Wen Zhang, Xin Li, Qian Chen, Zhuoru Jiang, Futao Chen, Xin Zhang, Wen-Wei Lin, Shing-Tung Yau, Bing Zhang

**Affiliations:** ^a^Department of Radiology, Nanjing Drum Tower Hospital, Affiliated Hospital of Medical School, Nanjing University, Nanjing 210008, China; ^b^School of Mathematics and Shing-Tung Yau Center, Southeast University, Nanjing 210096, China; ^c^Nanjing Center for Applied Mathematics, Nanjing 211135, China; ^d^Shanghai Institute for Mathematics and Interdisciplinary Sciences, Shanghai 200433, China; ^e^Department of Mathematics, National Taiwan Normal University, Taipei 116, Taiwan; ^f^Department of Neurosurgery, Nanjing Drum Tower Hospital, Affiliated Hospital of Medical School, Nanjing University, Nanjing 210008, China; ^g^Department of Diagnostic Radiology, Affiliated Jinling Hospital, Medical School of Nanjing University, Nanjing 210002, China; ^h^Department of Neurology, LuAn People’s Hospital, LuAn 237005, China; ^i^Yau Mathematical Sciences Center, Jingzhai, Tsinghua University, Beijing 100084, China; ^j^Medical Imaging Center, Nanjing Drum Tower Hospital, Affiliated Hospital of Medical School, Nanjing University, Nanjing 210008, China; ^k^Nanjing Key Laboratory for Cardiovascular Information and Health Engineering Medicine, Nanjing 210002, China; ^l^Institute of Medical Imaging and Artificial Intelligence, Nanjing University, Nanjing 210008, China

**Keywords:** glioma, OMT, SVD, deep learning

## Abstract

Glioma is the most common primary malignant brain tumor and preoperative genetic profiling is essential for the management of glioma patients. Our study focused on tumor regions segmentation and predicting the World Health Organization (WHO) grade, isocitrate dehydrogenase (IDH) mutation, and 1p/19q codeletion status using deep learning models on preoperative MRI. To achieve accurate tumor segmentation, we developed an optimal mass transport (OMT) approach to transform irregular MRI brain images into tensors. In addition, we proposed an algebraic preclassification (APC) model utilizing multimode OMT tensor singular value decomposition (SVD) to estimate preclassification probabilities. The fully automated deep learning model named OMT-APC was used for multitask classification. Our study incorporated preoperative brain MRI data from 3,565 glioma patients across 16 datasets spanning Asia, Europe, and America. Among these, 2,551 patients from 5 datasets were used for training and internal validation. In comparison, 1,014 patients from 11 datasets, including 242 patients from The Cancer Genome Atlas (TCGA), were used as independent external test. The OMT segmentation model achieved mean lesion-wise Dice scores of 0.880. The OMT-APC model was evaluated on the TCGA dataset, achieving accuracies of 0.855, 0.917, and 0.809, with AUC scores of 0.845, 0.908, and 0.769 for WHO grade, IDH mutation, and 1p/19q codeletion, respectively, which outperformed the four radiologists in all tasks. These results highlighted the effectiveness of our OMT and tensor SVD–based methods in brain tumor genetic profiling, suggesting promising applications for algebraic and geometric methods in medical image analysis.

## Significance

Accurate characterization of glioma is essential for effective clinical decision-making. Most current studies involve a limited number of patients and focus solely on single-gene tasks. This research introduces a novel deep learning model based on OMT and multimode tensor SVD to predict molecular markers using international multicenter datasets. Our approach efficiently compresses irrelevant information while enhancing tumor-region features through OMT. Additionally, we innovatively integrate an algebraic preclassification model, derived from multimode tensor SVD, with deep learning networks. This combination significantly improves the model’s ability to recognize tumor and classify genetic subtypes. Experimental validation on multicenter datasets demonstrates that our method is highly reproducible and generalizable, offering promising potential for glioma analysis and clinical applications.

Glioma is the most common primary malignant tumor in adults (1). The response to treatment and survival outcomes are influenced by the histological and genetic characteristics of gliomas, particularly tumor grade, isocitrate dehydrogenase (IDH) mutation status, and 1p/19q codeletion (2, 3). In 2021, the World Health Organization (WHO) 5th edition classification of central nervous system (CNS) tumors classified adult diffuse gliomas into three subgroups based on IDH mutation and 1p/19q codeletion status: glioblastoma, IDH-wildtype; astrocytoma, IDH-mutant, and 1p/19q-intact; and oligodendroglioma, IDH-mutant, and 1p/19q-codeleted (4). Numerous studies have shown that IDH-mutant gliomas are less aggressive and associated with a better prognosis than IDH-wildtype glioblastomas. Low-grade gliomas typically exhibit better treatment responses and more prolonged survival than high-grade gliomas. Therefore, accurately predicting the WHO grade, IDH mutation status, and 1p/19q-codeletion status of gliomas is crucial for informing prognosis and guiding treatment decisions (5).

Currently, histological and genetic information is obtained through the analysis of tumor tissue from surgery or biopsy, a costly and time-consuming process. For patients whose tumors cannot be safely resected, tumor samples may not be available, precluding further genetic analysis. As a result, there is an increasing demand for noninvasive methods that can provide crucial histological and genetic information about gliomas. MRI has become a key tool in preoperative diagnosis and evaluation of brain tumors (6–8). MRI can reveal glioma morphology and imaging heterogeneity within the tumor, which correlates with histological and genetic characteristics (9). With rapid advancements in computational technology, radiomics and deep learning techniques have gained widespread use in clinical decision-making systems, enhancing diagnosis and patient care across various clinical settings (10–12). Several pilot studies have explored the potential of deep learning models to predict genetic mutation status (13–15). However, these studies have been limited by small sample sizes (often fewer than 1,000 patients) and typically focus on predicting a single genetic feature at a time.

This study utilized a deep learning approach to predict WHO grade, IDH mutation status, and 1p/19q codeletion status based on tumor regions identified in preoperative MRI data. We incorporated preoperative two-dimensional (2D) or three-dimensional (3D) T_1_-weighted contrast-enhanced (T_1_CE) and T_2_-weighted imaging (T_2_WI) MRI data from 3,565 glioma patients across 16 datasets. However, the dataset presented challenges due to the mixture of 2D and 3D data and the lack of tumor labels in most cases, increasing the complexity of classification. We proposed the optimal mass transport (OMT) method to normalize multicenter datasets and segment the tumor region using deep learning to address these issues (16, 17). Unlike traditional techniques for brain tumor classification and segmentation, the OMT method achieves comprehensive normalization across multicenter datasets, offering superior generalization, robustness, and interpretability, making it suitable for integration with deep learning models.

A key technical challenge in deep learning for tumor region segmentation is the GPU memory limitation caused by the large size of MRI brain images. Traditional approaches, such as random cropping of raw brain images, often risk omitting critical information. We introduced the OMT technique to preserve the global structure of MRI data. OMT transforms MRI brain images into m × m × m tensors with minimal distortion, maintaining the overall 3D structure of the MRI data. The OMT density function enhances the tumor region while preserving the volume of nontumor regions within the OMT tensor, thereby improving segmentation performance.

This study utilized deep learning on OMT tumor regions to predict WHO grade, IDH mutation status, and 1p/19q codeletion status. We developed an algebraic preclassification (APC) model that leverages tumor region tensors embedded within OMT tensors and employs multimode tensor SVD for processing testing data. The multimode SVD offers an efficient and reliable algebraic preclassification model for probability estimation. By integrating the OMT technique with the APC model (OMT-APC) into a SE-ResNet model, we significantly improved the performance of the deep learning architecture. In contrast to traditional methods that focus on extracting radiomics features, statistical characteristics, or other information from tumor regions, the OMT-APC framework provides a more efficient and robust method for classification tasks.

## Result

### Baseline Patients’ Characteristics.

**Fig. 1.**
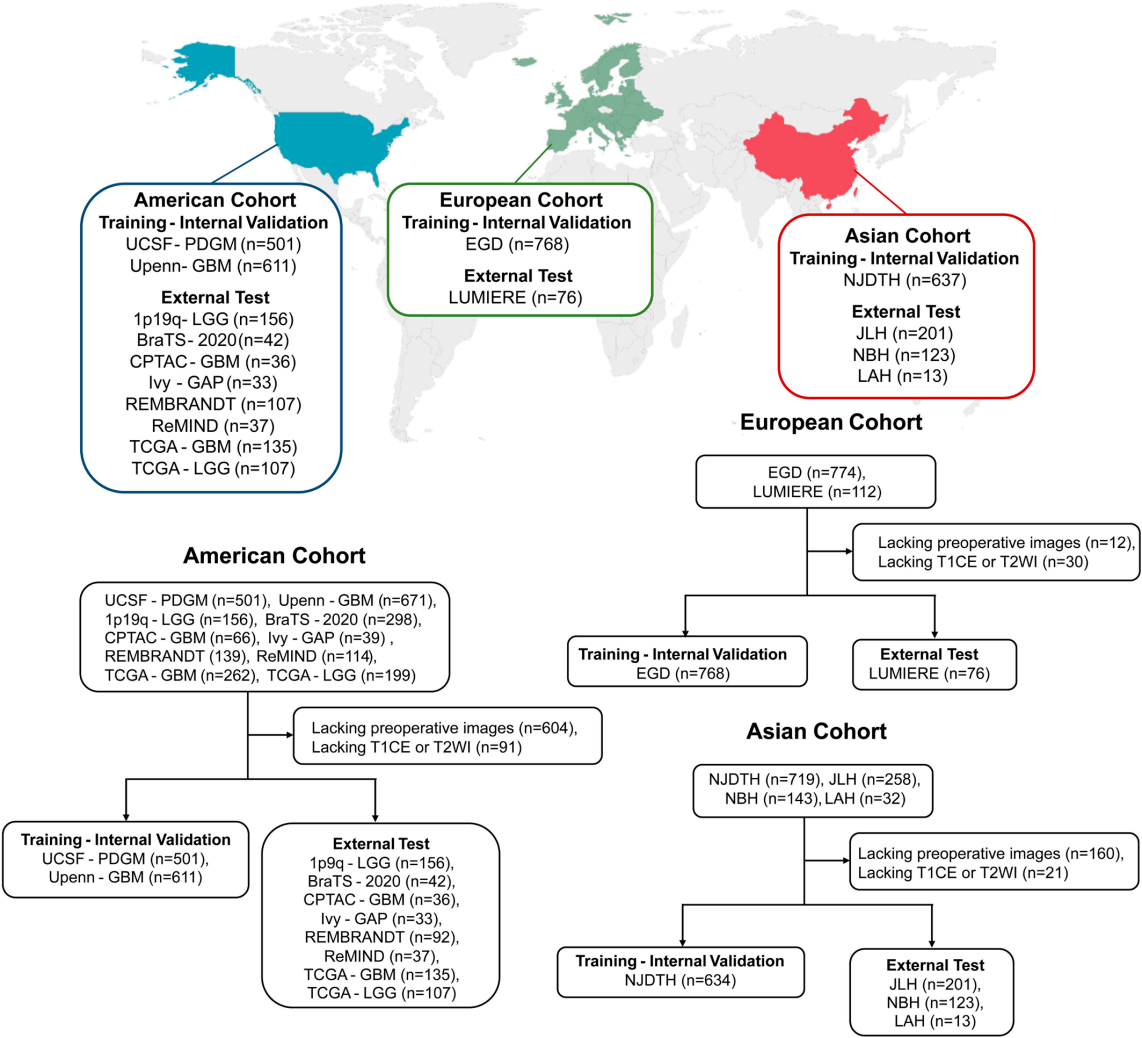
Dataset distribution and patient enrollment flowchart.

We enrolled a total of 3,565 patients in our study. The patients in the training set originated from 5 different datasets, and the test set data were collected from 11 different datasets, respectively. Table 1 illustrates a full overview of the baseline characteristics in the training and test set and Fig. 1 provides the inclusion–exclusion flowchart and the distribution of the patients from different datasets in the training set and test set.

**Table 1.** Baseline patients’ characteristics for the training-internal validation set and external test sets

	Total n = 3,565	Training-internal validation set n = 2,551	Independent external test set n = 1,014
Age	55.91 ± 14.79	57.22 ± 14.50	52.51 ± 15.00
Sex			
Female	1,468 (41.18%)	1,041 (40.81%)	427 (42.11%)
Male	2,016 (58.55)	1,509 (59.15%)	507 (50.00%)
Unknown	81 (2.27%)	1 (0.04%)	80 (7.89%)
IDH status			
Mutated	836 (23.45%)	498 (19.52%)	338 (33.33%)
Wildtype	2,026 (56.83%)	1,558 (61.07%)	468 (46.15%)
Unknown	703 (19.72%)	495 (19.40%)	208 (20.51%)
1p/19q codeletion status			
Codeleted	291 (8.16%)	144 (5.64%)	147 (14.50%)
Intact	488 (13.69%)	308 (12.07%)	180 (17.75%)
Unknown	2,786 (78.15%)	2,099 (82.28%)	687 (67.75%)
WHO Grade			
WHO Grade 2	596 (16.72%)	353 (13.84%)	243 (23.96%)
WHO Grade 3	341 (9.57%)	207 (8.11%)	134 (13.21%)
WHO Grade 4	2,613 (73.30%)	1,976 (77.46%)	637 (62.82%)
Unknown	15 (0.42%)	15 (0.59%)	0 (0.00%)
Histological diagnosis			
Glioblastoma	2,509 (70.38%)	1,902 (75.46%)	607 (59.86%)
Astrocytoma	578 (16.21%)	430 (16.86%)	148 (14.60%)
Oligodendroglioma	419 (11.75%)	160 (6.27%)	259 (25.54%)
Unknown	59 (1.65%)	59 (2.31%)	0 (0.00%)
Molecular diagnosis			
Glioblastoma, IDH-wildtype	2,026 (56.83%)	1,558 (61.07%)	468 (46.15%)
Glioblastoma, NOS	509 (14.28%)	384 (15.05%)	125 (12.33%)
Astrocytoma, IDH-mutated	506 (14.19%)	324 (12.70%)	182 (17.95%)
Astrocytoma, NOS	133 (3.73%)	68 (2.67%)	65 (6.41%)
Oligodendroglioma, IDH-mutated	291 (8.16%)	144 (5.64%)	147 (14.50%)
Oligodendroglioma, NOS	41 (1.15%)	14 (0.55%)	27 (2.66%)
Unknown	59 (1.65%)	59 (2.31%)	0 (0.00%)

IDH = Isocitrate Dehydrogenase; WHO = World Health Organization.

### Tumor Segmentation.

The BraTS 2023 Adult Glioma Challenge dataset (18–20) includes 1,251 labeled training brain images and 219 unlabeled brain images for online validation. We evaluated the performance of our OMT-based nnU-Net model for the whole tumor (WT), tumor core (TC), and enhanced tumor (ET) regions, comparing it with the top-performing models from the BraTS 2023 challenge. Specifically, we benchmarked our results against the winning model (21) and the third-place model (22), as summarized in Table 2. Notably, while the winner team employed generative adversarial networks and registration techniques to create an extensive dataset of 23,049 training samples, our approach—utilizing only 1,251 training samples with four density parameters—consistently delivered results that either outperformed or matched those researches.

**Table 2.** Lesion-wise Dice scores and lesion-wise Hausdorff distance 95 values of BraTS-2023 online validation

Method	Lesion-wise Dice score				Lesion-wise HD95			
	WT	TC	ET	Mean	WT	TC	ET	Mean
Winner	0.9101	0.8673	0.8509	0.8761	11.11	14.47	17.70	14.43
Third place	0.9063	0.8627	0.8432	0.8707	11.70	13.10	17.37	14.06
OMT nnU-Net	0.9082	0.8758	0.8566	0.8802	11.27	12.83	13.65	12.58

WT = Whole Tumor; TC = Tumor Core; ET = Enhancing Tumor.

### Model Capacity to Predict WHO Grade of Adult Diffuse Gliomas.

**Fig. 2.**
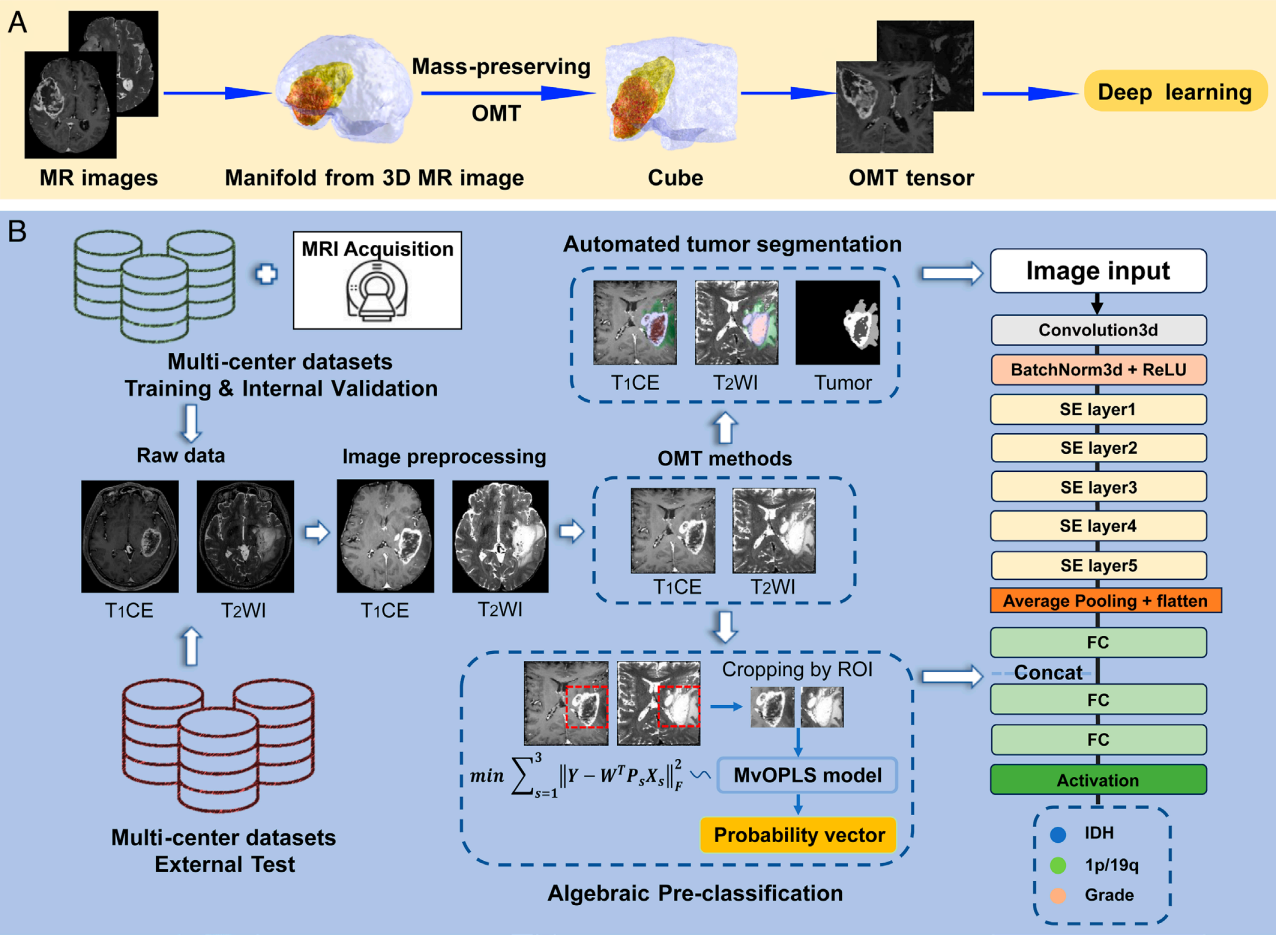
(*A*) OMT process of brain images. (*B*) Flowchart of the study design.

**Fig. 3.**
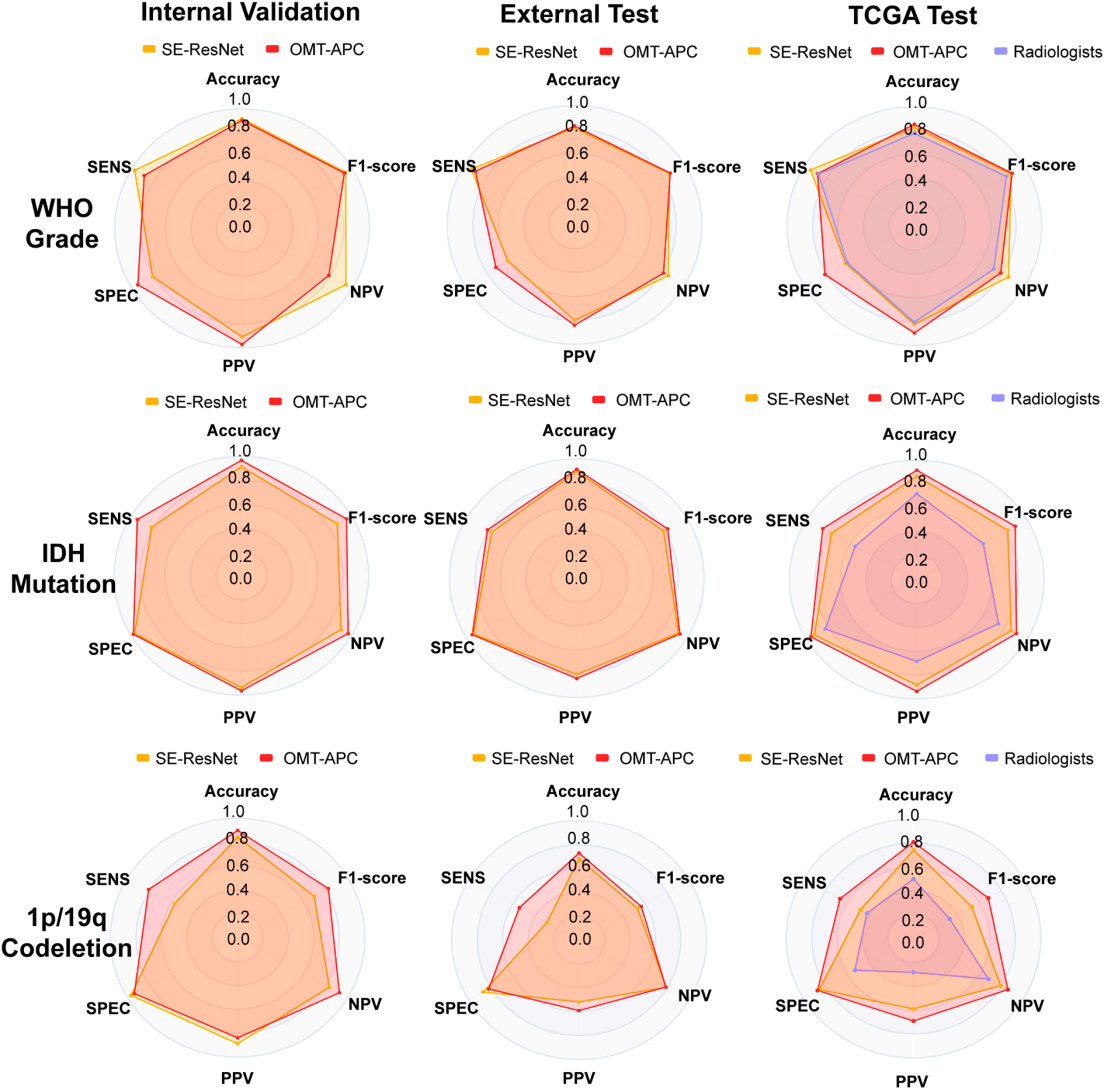
Radar maps comparing the performance of SE-ResNet Model, OMT-APC Model, and Radiologists in predicting WHO Grade, IDH mutation, and 1p/19q codeletion status.

On internal validation set, OMT-APC model achieved AUC of 0.917 and ACC of 0.906, and SE-ResNet model achieved AUC of 0.893 and ACC of 0.920. On the External test set, OMT-APC model achieved AUC of 0.807 and ACC of 0.830, and SE-ResNet model achieved AUC of 0.771 and ACC of 0.814. On TCGA test set, OMT-APC model achieved AUC of 0.845 and ACC of 0.855, and SE-ResNet model achieved AUC of 0.783 and ACC of 0.831. As shown in Table 3 and Fig. 2, we can observe that SE-ResNet outperformed MedTrans, Effnet, and PSPnet, and our APC method can enhance the performance of the SE-ResNet model in the WHO grade classification task. Through Fig. 3 and Table 3, we can observe that our method outperformed 4 radiologists.

**Table 3.** Performance of MedTransformer, Effnet, PSPnet, SE-ResNet model, OMT-APC model, and radiologists to predict WHO Grade of gliomas

Dataset	Model/reader	AUC (95% CI)	ACC	SENS	SPEC	PPV	NPV	F1-score
Internal validation	MedTrans	0.892 (0.862–0.922)	0.922	0.953	0.832	0.944	0.856	0.948
	Effnet	0.873 (0.841–0.906)	0.908	0.943	0.804	0.934	0.827	0.939
	PSPnet	0.892 (0.862–0.922)	0.913	0.934	0.850	0.949	0.812	0.941
	SE-ResNet	0.893 (0.851–0.934)	0.920	0.975	0.811	0.910	0.943	0.941
	OMT-APC	0.917 (0.891–0.942)	0.906	0.888	0.946	0.974	0.788	0.929
External test	MedTrans	0.767 (0.738–0.795)	0.807	0.923	0.610	0.800	0.824	0.857
	Effnet	0.758 (0.728–0.787)	0.801	0.926	0.589	0.792	0.825	0.854
	PSPnet	0.757 (0.728–0.786)	0.793	0.896	0.618	0.799	0.779	0.845
	SE-ResNet	0.771 (0.743–0.799)	0.814	0.937	0.605	0.800	0.851	0.863
	OMT-APC	0.807 (0.780–0.833)	0.830	0.900	0.714	0.841	0.808	0.869
TCGA test	MedTrans	0.771 (0.713–0.829)	0.818	0.930	0.612	0.816	0.825	0.869
	Effnet	0.759 (0.699–0.818)	0.806	0.917	0.600	0.809	0.797	0.860
	PSPnet	0.771 (0.713–0.829)	0.798	0.860	0.682	0.833	0.725	0.846
	SE-ResNet	0.783 (0.726–0.799)	0.831	0.943	0.624	0.822	0.855	0.878
	OMT-APC	0.845 (0.799–0.893)	0.855	0.879	0.812	0.896	0.784	0.887
	Radgiologist1	0.760 (0.701–0.819)	0.798	0.892	0.628	0.814	0.761	0.851
	Radgiologist2	0.705 (0.640–0.771)	0.745	0.841	0.570	0.781	0.662	0.810
	Radgiologist3	0.698 (0.631–0.764)	0.753	0.885	0.512	0.768	0.710	0.822
	Radgiologist4	0.799 (0.745–0.834)	0.819	0.866	0.733	0.855	0.750	0.861

AUC = area under the curve; ACC = accuracy; SENS = sensitivity; SPEC = specificity; PPV = positive predictive value; NPV = negative predictive value.

### Model Capacity to Predict IDH Mutation Status of Adult Diffuse Gliomas.

On internal validation set, the OMT-APC model achieved an AUC of 0.963 and ACC of 0.968, and the SE-ResNet model achieved an AUC of 0.892 and ACC of 0.915. On External test set, the OMT-APC model achieved an AUC of 0.879 and ACC of 0.913, and the SE-ResNet model achieved an AUC of 0.855 and ACC of 0.895. On TCGA test set, the OMT-APC model achieved an AUC of 0.908 and ACC of 0.917, and the SE-ResNet model achieved an AUC of 0.853 and ACC of 0.867. As shown in Table 4 and Fig. 2, we can observe that SE-ResNet outperformed MedTrans, Effnet, and PSPnet and our APC method can significantly enhance the performance of the SE-ResNet model in the IDH mutation status classification task. Through Fig. 3 and Table 4, we can observe that our method outperformed 4 radiologists.

**Table 4.** Performance of MedTransformer, Effnet, PSPnet, SE-ResNet model, OMT-APC model, and Radiologists to predict IDH mutation of gliomas

Dataset	Model/reader	AUC (95% CI)	ACC	SENS	SPEC	PPV	NPV	F1-score
Internal validation	MedTrans	0.867 (0.827–0.907)	0.886	0.801	0.932	0.867	0.895	0.833
	Effnet	0.878 (0.840–0.917)	0.903	0.795	0.962	0.921	0.895	0.853
	PSPnet	0.884 (0.846–0.922)	0.900	0.829	0.940	0.883	0.909	0.855
	SE-ResNet	0.892 (0.856–0.929)	0.915	0.815	0.970	0.937	0.905	0.871
	OMT-APC	0.963 (0.941–0.985)	0.968	0.945	0.981	0.965	0.970	0.955
External test	MedTrans	0.815 (0.772–0.858)	0.855	0.734	0.896	0.707	0.908	0.720
	Effnet	0.805 (0.761–0.848)	0.852	0.709	0.900	0.709	0.900	0.709
	PSPnet	0.846 (0.806–0.886)	0.869	0.797	0.894	0.720	0.928	0.757
	SE-ResNet	0.855 (0.816–0.894)	0.895	0.772	0.937	0.808	0.923	0.789
	OMT-APC	0.879 (0.843–0.915)	0.913	0.810	0.948	0.842	0.936	0.826
TCGA test	MedTrans	0.813 (0.752–0.873)	0.826	0.742	0.884	0.815	0.832	0.776
	Effnet	0.832 (0.774–0.890)	0.844	0.764	0.899	0.840	0.847	0.800
	PSPnet	0.839 (0.782–0.896)	0.849	0.787	0.891	0.833	0.858	0.809
	SE-ResNet	0.853 (0.813–0.883)	0.867	0.775	0.930	0.885	0.857	0.826
	OMT-APC	0.908 (0.863–0.952)	0.917	0.854	0.961	0.938	0.905	0.894
	Radgiologist1	0.582 (0.504–0.660)	0.633	0.303	0.860	0.600	0.642	0.403
	Radgiologist2	0.764 (0.697–0.830)	0.761	0.775	0.752	0.683	0.829	0.726
	Radgiologist3	0.664 (0.590–0.739)	0.702	0.461	0.868	0.707	0.700	0.558
	Radgiologist4	0.771 (0.705–0.837)	0.784	0.697	0.845	0.756	0.801	0.725

AUC = area under the curve; ACC = accuracy; SENS = sensitivity; SPEC = specificity; PPV = positive predictive value; NPV = negative predictive value.

### Model Capacity to Predict 1p/19q Codeletion Status of Adult Diffuse Gliomas.

On internal validation set, the OMT-APC model achieved an AUC of 0.873 and ACC of 0.9, and the SE-ResNet model achieved AUC of 0.771 and ACC of 0.844. On External test set, OMT-APC model achieved AUC of 0.681 and ACC of 0.73, and SE-ResNet model achieved AUC of 0.581 and ACC of 0.682. On TCGA test set, the OMT-APC model achieved AUC of 0.769 and ACC of 0.809, and the SE-ResNet model achieved an AUC of 0.668 and ACC of 0.742. As shown in Table 5 and Fig. 2, we can observe that SE-ResNet outperformed MedTrans, Effnet, and PSPnet and our APC method can significantly enhance the performance of the SE-ResNet model in the 1p/19q codeletion status classification task. Through Fig. 3 and Table 5, we can observe that our method outperformed 4 radiologists.

**Table 5.** Performance of MedTransformer, Effnet, PSPnet, SE-ResNet model, OMT-APC model, and Radiologists to predict 1p/19q codeletion of gliomas

Dataset	Model/reader	AUC (95% CI)	ACC	SENS	SPEC	PPV	NPV	F1-score
Internal validation	MedTrans	0.701 (0.588–0.831)	0.789	0.500	0.919	0.737	0.803	0.596
	Effnet	0.753 (0.638–0.869)	0.822	0.571	0.935	0.800	0.829	0.667
	PSPnet	0.684 (0.560–0.808)	0.767	0.464	0.903	0.684	0.789	0.553
	SE-ResNet	0.771 (0.671–0.898)	0.844	0.571	0.968	0.889	0.833	0.695
	OMT-APC	0.873 (0.810–0.944)	0.900	0.808	0.938	0.840	0.924	0.824
External test	MedTrans	0.493 (0.394–0.593)	0.608	0.167	0.820	0.308	0.672	0.216
	Effnet	0.525 (0.424–0.625)	0.628	0.229	0.820	0.379	0.689	0.286
	PSPnet	0.514 (0.414–0.614)	0.622	0.208	0.820	0.357	0.683	0.263
	SE-ResNet	0.581 (0.481–0.681)	0.682	0.292	0.870	0.519	0.719	0.373
	OMT-APC	0.681 (0.585–0.776)	0.730	0.542	0.820	0.591	0.788	0.565
TCGA test	MedTrans	0.565 (0.433–0.697)	0.685	0.259	0.871	0.467	0.730	0.333
	Effnet	0.621 (0.490–0.751)	0.719	0.370	0.871	0.556	0.761	0.444
	PSPnet	0.602 (0.471–0.733)	0.708	0.333	0.871	0.529	0.750	0.409
	SE-ResNet	0.668 (0.518–0.776)	0.742	0.481	0.855	0.591	0.791	0.530
	OMT-APC	0.769 (0.653–0.884)	0.809	0.667	0.871	0.692	0.857	0.679
	Radgiologist1	0.478 (0.346–0.609)	0.517	0.385	0.571	0.270	0.692	0.317
	Radgiologist2	0.543 (0.409–0.676)	0.640	0.308	0.778	0.364	0.731	0.333
	Radgiologist3	0.446 (0.317–0.576)	0.472	0.385	0.508	0.244	0.667	0.299
	Radgiologist4	0.443 (0.313–0.572)	0.371	0.615	0.270	0.258	0.630	0.364

AUC = area under the curve; ACC = accuracy; SENS = sensitivity; SPEC = specificity; PPV = positive predictive value; NPV = negative predictive value.

## Discussion

This study developed a deep learning OMT-APC model that integrates OMT and multimode tensor SVD to predict the WHO grade, IDH mutation, and 1p/19q codeletion status of adult diffuse gliomas using preoperative MR images.

The OMT method preserved the global structure of 3D MRI brain images while enlarging the tumor region within the OMT tensor. By varying parameters in the density function, OMT facilitated data augmentation and ensured reliable tumor segmentation (16, 17). By weighting the tumor region through the density function, we are able to amplify the spatial proportion of the tumor region on the tensor image, successfully enhancing the segmentation accuracy of TC and ET. In classification tasks, amplifying the tumor region also facilitates information extraction, helping the AI model deeply mine molecular genetic information in the images. Additionally, the OMT method standardized on multicenter imaging datasets can stably eliminate differences between data from different centers and exhibits strong robustness.

The truncated SVDs of the s-mode unfolding matrices (s = 1, 2, 3) of the OMT tensors, coupled with a multiview orthonormalized partial least squares optimization approach, provided an algebraic preclassification model. This model estimated the probabilities of preclassifications for testing data, enhancing classification accuracy. The APC model eliminated the need for neural network pretraining, providing preclassification data for deep learning tasks while significantly reducing computational time.

We trained our OMT-APC model on MRI data from 2,551 glioma patients across five centers. When tested on the publicly available TCGA dataset, the model achieved an AUC of 0.845 for WHO grade prediction, 0.908 for IDH mutation status prediction, and 0.769 for 1p/19q codeletion status prediction. Choi developed a deep convolutional neural network (CNN)–based deep learning model with 1166 glioma patients’ images and obtained an AUC of 0.86 on TCGA test set (23). Van der Voort developed another CNN model using a patient cohort of 1508 glioma patients and also tested on TCGA set, achieving an AUC of 0.81, 0.90, and 0.85 for WHO Grade, IDH mutation, and 1p/19q codeletion task, respectively (24). Our method outperformed previous research and obtained higher performance in multiclassification tasks on the same TCGA test set. This OMT-APC model enabled automated tumor segmentation and multitask genetic classification across diverse populations, MRI machine vendors, and imaging protocols, outperforming radiologists and demonstrating promising potential for broad clinical application.

The WHO CNS5 classification emphasizes the critical role of molecular diagnosis in adult diffuse gliomas (25). IDH mutation and 1p/19q codeletion status are key factors in glioma classification (26). IDH mutations are associated with a more favorable prognosis and better treatment response, regardless of histological presentation (27). Additionally, 1p/19q codeletion distinguishes oligodendroglioma from astrocytoma in IDH-mutated glioma patients (28). Identifying these molecular markers is essential for therapeutic decisions and clinical management (29).

Our OMT-APC model is particularly beneficial for patients where tumor resection is unsafe and burr-hole biopsy is performed solely for diagnostic purposes. In these cases, the model provides a noninvasive alternative. Furthermore, in situations where sample bias creates diagnostic uncertainty, the model can serve as an additional check for histological results.

Comparing with previous methods for the segmentation and classification of brain tumors, the OMT method can achieve complete normalization on multicenter datasets, demonstrating that generalization, robustness, and visibility which is suitable for the CNN model. The multimode SVD provides an efficient algebraic preclassification model for the probability estimation which can be used to significantly enhance classification accuracy. Moreover, our approach relies solely on 2D or 3D T_1_CE and T_2_WI images for segmentation and classification tasks without incorporating advanced modalities such as diffusion-weighted imaging, perfusion-weighted imaging, or susceptibility-weighted imaging. This highlights the strong generalizability of the OMT-APC method in medical imaging applications.

Future improvements will focus on enhancing the accuracy of the OMT method in segmenting the WT region. The research will focus on identifying the optimal density function within OMT by leveraging machine learning to maximize the extraction of information from suspected tumor regions in MR images, thereby enhancing edge recognition of the WT region. The OMT-APC approach incorporates both geometric transformations and algebraic features of MR images, enabling effective tumor segmentation and classification. Consequently, future efforts will also aim to develop advanced algebraic and geometric techniques to extract feature information from other medical imaging modalities, supporting image-based gene classification, medical diagnosis, and treatment planning.

## Materials and Methods

### Data Collection.

We enrolled a total of 3565 patients in our study and conducted a retrospective analysis by gathering preoperative contrast-enhanced T_1_CE and T_2_WI from adult diffuse gliomas patients undergoing surgery or biopsy. This research was approved by the Medical Ethical committee and the Institutional Review Board of Nanjing Drum Tower Hospital (2022-364-02) and registered on Clinical Trial (NCT05624736). The written informed consent was waived due to the retrospective nature of this study.

Four private datasets from China between January 2018 and December 2023 included Nanjing Drum Tower Hospital (NJDTH), Jinling Hospital (JLH), Nanjing Brain Hospital (NBH), and LuAn People’s Hospital (LAH). Two publicly available datasets from Europe included the Erasmus Glioma database (EGD) dataset (30) and the LUMIERE dataset (31). Ten publicly available datasets from America included the University of Pennsylvania glioblastoma (UPenn-GBM) dataset (32), the University of California San Francisco Preoperative Diffuse Glioma MRI Dataset (UCSF-PDGM) dataset (33), the ReMIND dataset (34), the Ivy Glioblastoma Atlas (Ivy-GAP) dataset (35), the Rembrandt brain cancer dataset (REMBRANDT) (36), the National Cancer Institute Clinical Proteomic Tumor Analysis Consortium Glioblastoma Multiforme (CPTAC-GBM) dataset, the Low grade glioma 1p19qDeletion (1p19q-LGG) dataset (37), the BraTS2020 dataset, The Cancer Genome Atlas Glioblastoma Multiforme (TCGA-GBM) dataset, and the Cancer Genome Atlas Low Grade Glioma (TCGA-LGG) dataset (18). The cases in the BraTS2020 that overlap with TCGA-GBM and TCGA-LGG were excluded. NJDTH, EGD, Upenn-GBM, UCSF-PDGM and ReMIND were designated as training and internal validation set. JLH, NBH, LAH, LUMIERE, Ivy-GAP, REMBRANDT, CPTAC-GBM, 1p19q-LGG, BraTS2020, TCGA-GBM, and TCGA-LGG were designated as external validation set.

Our inclusion criteria were as follows: i) newly diagnosed and pathologically confirmed glioma. ii) available T_1_CE and T_2_WI images within 2 wk before surgery or biopsy. iii) Known WHO grade, IDH mutation or 1p/19q codeletion status. Exclusion criteria included i) lacking preoperative images; ii) lacking T_1_CE or T_2_WI sequence. Therefore, a total of 2551 patients were included in the training and internal validation set with a ratio of 4:1, and 1014 patients were included in the external test set.

### Image Processing and OMT.

The image data used in this study were sourced from a 16-center dataset and underwent various preprocessing methods due to the lack of raw MRI data for standardized processing. The raw images were converted to standardized skull-stripped brain images through the following steps:

1. Resampling: Both 2D and 3D T_2_WI and T_1_CE were resampled to a standard 3D voxel space with a resolution of 1×1×1 ${mm}^{3}$.
2. Intensity Normalization: Image grayscale values were normalized to the range [0, 1,000].
3. Bias Field Correction: Bias field distortions were corrected.
4. Image Registration: T_2_WI was used as the reference image, and T_1_CE images were rigidly coregistered to the T_2_WI modality (38, 39).
5. Brain Extraction: A combination of BET and neural network models was applied to extract the brain region, removing the skull, eyes, nose, and neck to mitigate their impact on brain image analysis (40).

Finally, the preprocessed images were further analyzed using OMT techniques.

Consider a discrete simplicial manifold M with a tetrahedral mesh representation of the above 3D brain image. This manifold comprises sets of vertices V(M) and tetrahedrons $T\left(M\right)$. Let $H_{e}\in \left[0,1\right]$ represent the normalized grayscale values derived from contrast-enhanced histogram equalization of the brain image, and let $H_{e}\left(v\right)$ denote the grayscale value at vertex v. Denote WT region as $R_{w}$. A density function $\rho_{\gamma }$ at v is defined as follows:

[1] $$\rho_{\gamma }\left(v\right)=\left\{\begin{array}{cc} \exp\left(\gamma \cdot H_{e}\left(v\right)\right), & ifv\in R_{w}\\ 1, & otherwise \end{array}\right.,$$

where γ>0 is a hyperparameter. We further define $\rho_{\gamma }$ at tetrahedron $\tau \in T\left(M\right)$ and the local volume measure $m_{\rho_{\gamma }}$ at vertex v∈V(M) by $\rho_{\gamma }\left(\tau \right)=\frac{1}{4}\sum_{\tau \in N\left(v\right)}\rho_{\gamma }\left(v\right)$ and $m_{\rho_{\gamma }}\left(v\right)=\frac{1}{4}\sum_{\tau \in N\left(v\right)}\rho_{\gamma }\left(v\right)\left|\tau \right|$, where N(v) is the set of 1-ring neighboring tetrahedrons of v, and $\left|\tau \right|$ represents the volume of tetrahedron τ.

Using $\rho_{\gamma }$, a mass-preserving discrete OMT map $f_{\rho_{\gamma }}^{*}:M\to B^{3}$ is proposed, as detailed in (17, 41, 42), to transform M into a discrete unit sphere $B^{3}=\left\{x\in R^{3}:\;∥x{∥}_{2}\leq 1\right\}$. This map is defined by

$$f_{\rho_{\gamma }}^{*}=\underset{f_{\rho_{\gamma }}\in F_{\rho_{\gamma }}}{\text{argmin}}\sum_{v\in V(M)}∥v-f_{\rho_{\gamma }}\left(v\right){∥}_{2}^{2}m_{\rho_{\gamma }}(v),$$

where the set of feasible maps $F_{\rho_{\gamma }}$ is

$$F_{\rho_{\gamma }}=\left\{f_{\rho_{\gamma }}:M\to B^{3}|\rho_{\gamma }\left(\tau \right)\left|\tau \right|=f_{\rho_{\gamma }}\left(\tau \right),\forall \tau \in T\left(M\right)\right\}.$$

Similarly, the discrete solid cube C, consisting of vertices V(C) and tetrahedrons $T\left(C\right)$, can be transformed into $B^{3}$ using volume-preserving OMT map $f^{*}$:

$$f^{*}=\underset{f\in F}{\text{argmin}}\sum_{v\in V\left(C\right)}∥v-f\left(v\right){∥}_{2}^{2}\left(\frac{1}{4}\sum_{\tau \in N\left(v\right)}\left|\tau \right|\right),$$

where

$$F=\left\{f:C\to B^{3}|\left|\tau \right|=f\left(\tau \right),\forall \tau \in T\left(C\right)\right\}.$$

By composing these maps, ${\hat{f}}_{\rho_{\gamma }}^{*}={\left(f^{*}\right)}^{-1}\circ f_{\rho_{\gamma }}^{*}$ enables the transformation of the irregular domain M into the solid cube C, preserving mass. The resulting 3D OMT tensor derived from C can serve as input data for deep learning models.

**Fig. 4.**
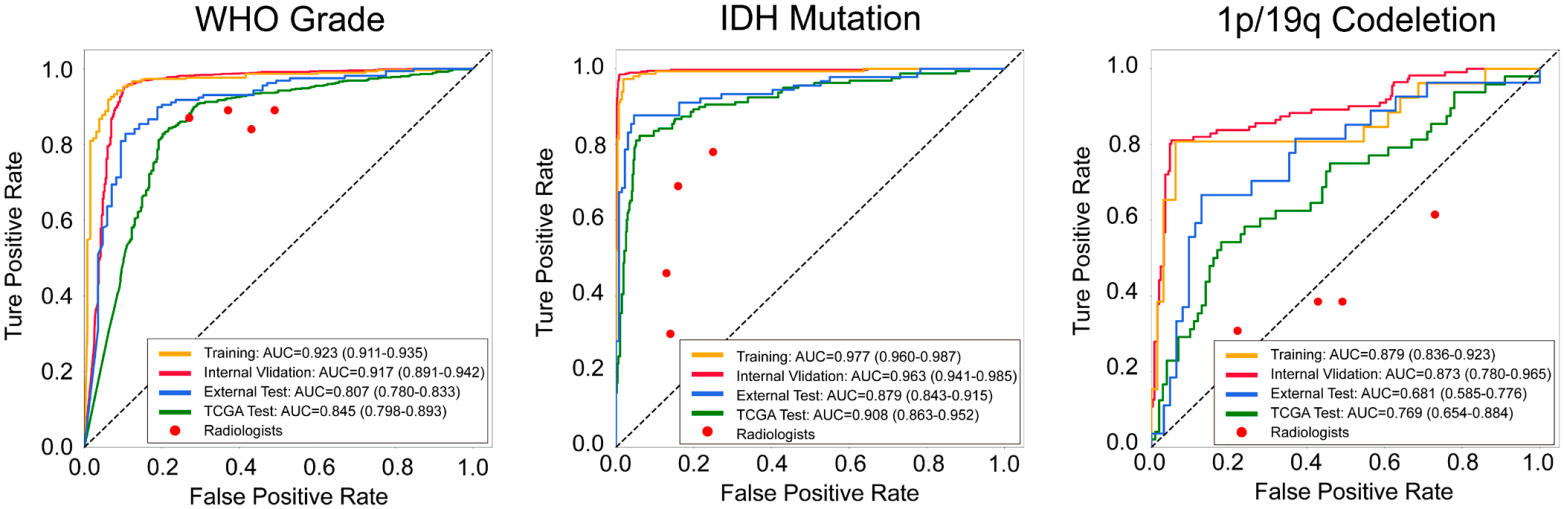
ROC curves for OMT-APC model and Radiologists in predicting WHO Grade, IDH mutation, and 1p/19q codeletion status.

As illustrated in Fig. 4, the OMT map transforms the irregular brain image into an m×m×m tensor with minimal distortion, preserving the global structure of the 3D MR image. The density function $\rho_{\gamma }$ defined in Eq. **1** enhances the tumor region while maintaining the volume of the nontumor region in the OMT tensor. Furthermore, varying the parameter γ allows for data augmentation, increasing diversity and mitigating overfitting during model training.

### Automated Tumor Segmentation.

In this study, the fully automated nnU-Net (43) was utilized for model training, incorporating fivefold cross-validation for each network. This updated version of nnU-Net includes a comprehensive preprocessing and data augmentation pipeline. To further enhance the training data, we augment it from $n_{t}$ to $4n_{t}$ using OMT tensors with values of γ=1.0,1.5,1.75,2.0. To accommodate this increase in data, the network size is adjusted asymmetrically, doubling the number of filters in the encoder while maintaining the number of filters in the decoder. The maximum number of filters is also increased to 512. Additionally, all instance normalization layers are replaced with group normalization layers, setting the number of groups to 32.

### Algebraic Preclassification Model.

Let ${\hat{T}}_{i}\in R^{\hat{m}\times \hat{m}\times \hat{m}}$ for i=1,⋯,n represent the OMT image tensors with γ of 1.75 in the training data. Let $y_{i}$ denote the associated label vector, which can be either ${\left[0,1\right]}^{T}$ or ${\left[1,0\right]}^{T}$. Define $n_{1}$ and $n_{2}$ as the number of instances where $y_{i}={\left[0,1\right]}^{T}$ and $y_{i}={\left[1,0\right]}^{T}$, respectively, so that $n=n_{1}+n_{2}$.

For each tensor ${\hat{T}}_{i}$, we extract a smaller tensor $T_{i}\in R^{m\times m\times m}$ corresponding to the WT region. Using these tumor region tensors $T_{i}$ for i=1,⋯,n, we propose a preclassification model that estimates the probability function for the testing data. These estimated probabilities can then be incorporated into the training model to improve classification accuracy.

Define the s-mode unfolding matrix of $T_{i}$ as ${T_{i}}^{\left(s\right)}\in R^{m\times m^{2}}$ for s=1,2,3. We approximate ${T_{i}}^{\left(s\right)}$ using the truncated SVD:

$${T_{i}}^{\left(s\right)}\approx {{U_{i}}^{\left(s\right)}{\Sigma_{i}}^{\left(s\right)}\left({V_{i}}^{\left(s\right)}\right)}^{T},$$

where ${\Sigma_{i}}^{\left(s\right)}\in R^{j\times j}$ with j<m. We then vectorize ${U_{i}}^{\left(s\right)}{\Sigma_{i}}^{\left(s\right)}$ to obtain $x_{i}^{\left(s\right)}=vec\left({U_{i}}^{\left(s\right)}{\Sigma_{i}}^{\left(s\right)}\right)\in R^{d}$, where d=m×j. We define the matrices $X={\left[X_{1}^{T},X_{2}^{T},X_{3}^{T}\right]}^{T}\in R^{3d\times n}$ with $X_{s}=\left[{x_{1}}^{\left(s\right)}\cdot \cdot \cdot {x_{n}}^{\left(s\right)}\right]D_{n}\in R^{d\times n}$ and $Y=\left[y_{1}\cdot \cdot \cdot y_{1}\right]D_{n}\in R^{2\times n}$, where $D_{n}=diag\left(d_{1},\cdots ,d_{n}\right)$ with $d_{i}=\frac{1}{\sqrt{n_{1}}}$, if $y_{i}={\left[1,0\right]}^{T}$; $d_{i}=\frac{1}{\sqrt{n_{2}}}$, if $y_{i}={\left[0,1\right]}^{T}$. This weighting matrix $D_{n}$ balances the contributions of the two label classes during the optimization in Eq. **2** below.

We then formulate a multiview orthonormalized partial least squares (MvOPLS) optimization problem, incorporating a Tikhonov regularize:

[2] $$\underset{\left\{P_{s}\in R^{d\times k}\right\},\;W\in R^{k\times 2}}{\text{min}}\left(\sum_{s=1}^{3}∥Y-W^{T}P_{s}^{T}X_{s}{∥}_{F}^{2}+\sum_{s=1}^{3}\gamma_{s}∥P_{s}W{∥}_{F}^{2}\right).$$

Let $P={\left[{P_{1}}^{T},{P_{2}}^{T},{P_{3}}^{T}\right]}^{T},$ $A=XY^{T}YX^{T}\in R^{3d\times 3d},$ and $B=diag\left(X_{1}X_{1}^{T},X_{2}X_{2}^{T},X_{3}X_{3}^{T}\right)$$+diag\left(\gamma_{1}I_{d},\gamma_{2}I_{d},\gamma_{3}I_{d}\right).$ As the derivations in (44), the optimizer $P_{*}$ of Eq. **2** can be found by solving the generalized eigenvalue problem:

$$\underset{P\in R^{3d\times k}}{\text{max}}tr\left(P^{T}AP\right),\;\;\;\;\;\mathrm{subject}\;\mathrm{to}\;\;\;\;\;P^{T}BP=I_{k},$$

where $P_{*}$ consists of the k normalized eigenvectors of the matrix pair (A,B) corresponding to the k largest eigenvalues. Once $P_{*}\equiv {\left[P_{*1}^{T},P_{*2}^{T},P_{*3}^{T}\right]}^{T}$ is computed, we can obtain the optimizer $W_{*}$ for Eq. **2**:

$$W_{*}={\left(P_{*}^{T}BP_{*}\right)}^{-1}P_{*}^{T}XY^{T}.$$

For the testing data, we have a 3D OMT image tensor ${\hat{T}}_{t}\in R^{\hat{m}\times \hat{m}\times \hat{m}}$. We use an OMT-based nnU-Net model to segment the tumor region in ${\hat{T}}_{t}$, and then crop the corresponding tensor $T_{t}\in R^{m\times m\times m}$. Using the pretrained MvOPLS model, we compute the probability approximations for the preclassification of ${\hat{T}}_{t}$:

$$y_{t}^{\left(s\right)}=W_{*}^{T}P_{*s}^{T}x_{t}^{\left(s\right)},s=1,2,3,$$

where $x_{t}^{\left(s\right)}$ is the vectorization of the truncated SVD of the s-mode unfolding matrix of $T_{t}$. For each patient, this results in six algebraic probability preclassifications based on the two modalities (T_1_CE and T_2_WI). The Tikhonov regularization parameters are typically set to $10^{-4}$.

### SE-ResNet with Algebraic Preclassification (APC-SE-ResNet).

The middle and right sections of Fig. 4*B* depict the SE-ResNet architecture(45) integrated with a pretrained MvOPLS model for the classification tasks of predicting the IDH status, 1p/19q codeletion, and WHO grade. The top-middle blue box shows that the SE-ResNet architecture uses OMT tensors from T_1_CE, T_2_WI, and tumor labels as inputs. In the bottom-middle blue box, the architecture concatenates the probability vectors generated by the MvOPLS preclassification model with the output vector from the fully connected (FC) layer. This concatenated vector is subsequently passed onto the next FC layer for further processing.

Due to the class imbalance in the IDH, 1p/19q, and WHO grade tasks, we use cross-entropy loss as the training loss function. To address the imbalance issue, we also apply label-smoothing regularization to adjust the ground-truth distribution:

$$\hat{y}=\left(1-\epsilon \right)y+\frac{\epsilon }{C},$$

where ϵ is the label-smoothing parameter and C is the number of classes. For our classification tasks, we set ϵ=0.1.

We adopt the AdaMax optimizer, a variant of Adam using the infinity norm. The initial learning rate is $10^{-5}$ and decreases by a factor of 0.96 every 10 epochs. The model is trained for up to 500 epochs.

Fig. 4 shows the entire workflow of our proposed method, the OMT-APC model, for predicting WHO grade, IDH mutation, and 1p/19q codeletion. The OMT-APC model process includes the following steps:

1. Image Preprocessing: Extracting brain images from raw data.
2. Tumor Segmentation: Using an OMT-based nnU-Net model to segment tumor regions in the OMT tensors of the extracting brain images.
3. Preclassification: Applying a pretrained MvOPLS model to generate algebraic preclassifications of the testing OMT tensors.
4. Model Training: Training the OMT-APC model using both the training OMT tensors and the algebraic preclassifications of the test data.

For the WHO grade classification, low-grade gliomas (WHO grade 2 and 3) are considered the positive class, while high-grade gliomas (WHO grade 4) are the negative class. For IDH mutation status classification, IDH-mutated samples are treated as the positive class. Similarly, for 1p/19q codeletion status classification, 1p/19q codeleted samples are regarded as the positive class. The TCGA-GBM and TCGA-LGG datasets serve as benchmarks for evaluating the classification model.

### Model Evaluation and Human–AI competing Test.

For comparison, we chose the following three deep learning models widely utilized in medical imaging: MedTrans (46), Effnet (47), and PSPnet (48).

The performance of the deep learning model was evaluated by assessing the AUC of the receiver operative characteristic curve (ROC), Accuracy (ACC), Sensitivity (SENS), Specificity (SPEC), Positive Predictive Value (PPV), Negative Predictive Value (NPV), and F1-score.

TCGA-GBM and TCGA-LGG datasets were designated as test set for human–AI competing because these two datasets were most widely used in machine learning–based Glioma imaging genotyping research. Four board-certificated neuroradiologists, including two junior neuroradiologists with 3-y-experience in glioma imaging and two senior neuroradiologist with 8 y of experience, independently read T_1_CE and T_2_WI images and predicted the WHO grade, IDH mutation, and 1p/19q codeletion status of patients. The radiologists were blinded to clinical characteristics and pathological results during the evaluation.

## Data Availability

The MRI data for American cohort and European cohort are available online with the following links: UCSF-PDGM (49): https://www.cancerimagingarchive.net/collection/ucsf-pdgm/.Upenn-GBM (50): https://www.cancerimagingarchive.net/collection/upenn-gbm/. 1p/19q-LGG (51): https://www.cancerimagingarchive.net/collection/lgg-1p19 qdeletion/. BraTS-2020 (52): https://www.kaggle.com/datasets/awsaf49/brats20-dataset-training-validation. CPTAC-GBM (53): https://www.cancerimagingarchive.net/collection/cptac-gbm/. Ivy-GAP (54): http://cancerimagingarchive.net/collection/ivygap/. REMBRANDT (55): https://www.cancerimagingarchive.net/collection/rembrandt/. ReMIND (56): https://www.cancerimagingarchive.net/collection/remind/. TCGA-GBM (57): https://www.cancerimagingarchive.net/collection/tcga-gbm/. TCGA-LGG (58): https://www.cancerimagingarchive.net/collection/tcga-lgg/. EGD (59): https://xnat.health-ri.nl/REST/projects/egd. LUMIERE (60): https://doi.org/10.6084/m9.figshare.c.5904905.v1. The MRI data for Asian cohort are available with the request to the corresponding authors (zhangxin@njglyy.com; zhangbing_nanjing@nju.edu.cn). All the code developed and used throughout this research has been made open source and is available on Github (https://github.com/happywind-boy/OMT-APC) (61).

## References

[1] M. Price , CBTRUS statistical report: American Brain Tumor Association & NCI neuro-oncology branch adolescent and young adult primary brain and other central nervous system tumors diagnosed in the United States in 2016–2020. Neuro. Oncol. **26**, iii1–iii53 (2024). 38709657 10.1093/neuonc/noae047 PMC11073545

[2] H. Pinson , Epidemiology and survival of adult-type diffuse glioma in Belgium during the molecular era. Neuro Oncol. **26**, 191–202 (2024). 37651614 10.1093/neuonc/noad158 PMC10768998

[3] T. Nakase , Genome-wide polygenic risk scores predict risk of glioma and molecular subtypes. Neuro-oncology **26**, 1933–1944 (2024). 38916140 10.1093/neuonc/noae112 PMC11448969

[4] D. N. Louis , The 2021 WHO classification of tumors of the central nervous system: A summary. Neuro-Oncol **23**, 1231–1251 (2021). 34185076 10.1093/neuonc/noab106 PMC8328013

[5] T. Jiang , Clinical practice guidelines for the management of adult diffuse gliomas. Cancer Lett. **499**, 60–72 (2021). 33166616 10.1016/j.canlet.2020.10.050

[6] J. Zhou , Review of tracer kinetic models in evaluation of gliomas using dynamic contrast-enhanced imaging. Front. Oncol. **14**, 1380793 (2024). 38947892 10.3389/fonc.2024.1380793 PMC11211364

[7] S. S. Ahn , Identification of magnetic resonance imaging features for the prediction of molecular profiles of newly diagnosed glioblastoma. J. Neurooncol. **154**, 83–92 (2021). 34191225 10.1007/s11060-021-03801-y

[8] K. M. Kang , MRI scoring systems for predicting isocitrate dehydrogenase mutation and chromosome 1p/19q codeletion in adult-type diffuse glioma lacking contrast enhancement. Radiology **311**, e233120 (2024). 38713025 10.1148/radiol.233120

[9] A. Lasocki , Correlating MRI features with additional genetic markers and patient survival in histological grade 2–3 IDH-mutant astrocytomas. Neuroradiology **65**, 1215–1223 (2023). 37316586 10.1007/s00234-023-03175-0 PMC10338396

[10] S. Chen , Predicting MGMT promoter methylation in diffuse gliomas using deep learning with radiomics. J. Clin. Med. **11**, 3445 (2022). 35743511 10.3390/jcm11123445 PMC9224690

[11] H. Yang , Quantitative and qualitative parameters of DCE-MRI predict *CDKN2A/B* homozygous deletion in gliomas. Acad. Radiol. **31**, 3355–3365 (2024). 38443208 10.1016/j.acra.2024.02.017

[12] Z. Zhu , Radiomics for predicting grades, isocitrate dehydrogenase mutation, and oxygen 6-methylguanine-DNA methyltransferase promoter methylation of adult diffuse gliomas: Combination of structural MRI, apparent diffusion coefficient, and susceptibility-weighted imaging. Quant. Imaging Med. Surg. **14**, 9276–9289 (2024). 39698654 10.21037/qims-24-1110 PMC11652054

[13] G. Salle , Accuracy of radiomics in predicting IDH mutation status in diffuse gliomas: A bivariate meta-analysis. Radiol. Artif. Intell. **6**, e220257 (2024). 38231039 10.1148/ryai.220257 PMC10831518

[14] Y. Sha , Prediction of the molecular subtype of IDH mutation combined with MGMT promoter methylation in gliomas via radiomics based on preoperative MRI. Cancers (Basel) **15**, 1440 (2023). 36900232 10.3390/cancers15051440 PMC10001198

[15] H. Zhang , Deep learning radiomics for the assessment of telomerase reverse transcriptase promoter mutation status in patients with glioblastoma using multiparametric MRI. J. Magn. Reson. Imaging **58**, 1441–1451 (2023). 36896953 10.1002/jmri.28671

[16] W.-W. Lin , A novel 2-phase residual U-net algorithm combined with optimal mass transportation for 3D brain tumor detection and segmentation. Sci. Rep. **12**, 6452 (2022). 35440793 10.1038/s41598-022-10285-x PMC9018750

[17] W.-W. Lin , 3D brain tumor segmentation using a two-stage optimal mass transport algorithm. Sci. Rep. **11**, 14686 (2021). 34376714 10.1038/s41598-021-94071-1 PMC8355223

[18] S. Bakas, , Advancing the Cancer Genome Atlas glioma MRI collections with expert segmentation labels and radiomic features. Sci. Data **4**, 170117 (2017). 28872634 10.1038/sdata.2017.117 PMC5685212

[19] B. H. Menze , The multimodal brain tumor image segmentation benchmark (BRATS). IEEE Trans. Med. Imaging **34**, 1993–2024 (2015). 25494501 10.1109/TMI.2014.2377694 PMC4833122

[20] U. Baid, , The RSNA-ASNR-MICCAI BraTS 2021 benchmark on brain tumor segmentation and radiogenomic classification. arXiv [Preprint] (2021), 10.48550/arXiv.2107.02314 (Accessed 12 September 2021).

[21] A. Ferreira, , How we won BraTS 2023 adult glioma challenge? Just faking it! Enhanced Synthetic Data Augmentation and Model Ensemble for brain tumour segmentation. arXiv [Preprint] (2024), 10.48550/arXiv.2402.17317 (Accessed 17 July 2024).

[22] F. Maani, , Advanced tumor segmentation in medical imaging: An ensemble approach for BraTS 2023 adult glioma and pediatric tumor tasks. arXiv {Preprint] (2024), 10.48550/arXiv.2403.09262 (Accessed 14 March 2024).

[23] Y. S. Choi , Fully automated hybrid approach to predict the IDH mutation status of gliomas via deep learning and radiomics. Neuro Oncol. **23**, 304–313 (2021). 32706862 10.1093/neuonc/noaa177 PMC7906063

[24] S. R. van der Voort , Combined molecular subtyping, grading, and segmentation of glioma using multi-task deep learning. Neuro Oncol. **25**, 279–289 (2023). 35788352 10.1093/neuonc/noac166 PMC9925710

[25] C. Horbinski, , Molecular testing for the World Health Organization classification of central nervous system tumors: A review. JAMA Oncol. **11**, 317–328 (2024). 10.1001/jamaoncol.2024.5506 PMC12344474 39724142

[26] A. Azizova , Preoperative prediction of diffuse glioma type and grade in adults: A gadolinium-free MRI-based decision tree. Eur. Radiol. **35**, 1242–1254 (2024). 39425768 10.1007/s00330-024-11140-5 PMC11836213

[27] H. Yan , IDH1 and IDH2 mutations in gliomas. N. Engl. J. Med. **360**, 765–773 (2009). 19228619 10.1056/NEJMoa0808710 PMC2820383

[28] J. E. Eckel-Passow , Glioma Groups Based on 1p/19q, IDH, and TERT Promoter Mutations in Tumors. N. Engl. J. Med. **372**, 2499–2508 (2015). 26061753 10.1056/NEJMoa1407279 PMC4489704

[29] M. I. Fuente, , The role of vorasidenib in the treatment of isocitrate dehydrogenase-mutant glioma. Neuro Oncol. (2024), 10.1093/neuonc/noae259. PMC12187374 39723472

[30] S. R. van der Voort , The Erasmus Glioma Database (EGD): Structural MRI scans, WHO 2016 subtypes, and segmentations of 774 patients with glioma. Data In Brief **37**, 107191 (2021). 34159239 10.1016/j.dib.2021.107191 PMC8203723

[31] Y. Suter , The LUMIERE dataset: Longitudinal glioblastoma MRI with expert RANO evaluation. Sci. Data **9**, 768 (2022). 36522344 10.1038/s41597-022-01881-7 PMC9755255

[32] S. Bakas , The University of Pennsylvania glioblastoma (UPenn-GBM) cohort: Advanced MRI, clinical, genomics, & radiomics. Sci. Data **9**, 453 (2022). 35906241 10.1038/s41597-022-01560-7 PMC9338035

[33] E. Calabrese , The University of California San Francisco preoperative diffuse glioma MRI dataset. Radiol. Artif. Intell. **4**, e220058 (2022). 36523646 10.1148/ryai.220058 PMC9748624

[34] P. Juvekar , ReMIND: The brain resection multimodal imaging database. Sci. Data **11**, 494 (2024). 38744868 10.1038/s41597-024-03295-z PMC11093985

[35] R. B. Puchalski , An anatomic transcriptional atlas of human glioblastoma. Science **360**, 660–663 (2018). 29748285 10.1126/science.aaf2666 PMC6414061

[36] Y. Gusev , The REMBRANDT study, a large collection of genomic data from brain cancer patients. Sci. Data **5**, 180158 (2018). 30106394 10.1038/sdata.2018.158 PMC6091243

[37] Z. Akkus , Predicting deletion of chromosomal arms 1p/19q in low-grade gliomas from MR images using machine intelligence. J. Digit. Imaging **30**, 469–476 (2017). 28600641 10.1007/s10278-017-9984-3 PMC5537096

[38] K. Marstal, F. Berendsen, M. Staring, S. Klein, “SimpleElastix: A user-friendly, multi-lingual library for medical image registration” in Proceedings of the IEEE conference on computer vision and pattern recognition workshops (2016), pp 134–142.

[39] K. Ntatsis , “itk-elastix: Medical image registration in Python” in Proceedings of the 22nd Python in Science Conference (2023), pp 101–105.

[40] S. M. Smith, Fast robust automated brain extraction. Hum. Brain Mapp. **17**, 143–155 (2002). 12391568 10.1002/hbm.10062 PMC6871816

[41] T.-M. Huang, W.-H. Liao, W.-W. Lin, M.-H. Yueh, S.-T. Yau, Convergence analysis of volumetric stretch energy minimization and its associated optimal mass transport. SIAM J. Imaging Sci. **16**, 1825–1855 (2023).

[42] M.-H. Yueh, T.-M. Huang, T. Li, W.-W. Lin, S.-T. Yau, Projected gradient method combined with homotopy techniques for volume-measure-preserving optimal mass transportation problems. J. Sci. Comput. **88**, 64 (2021).

[43] F. Isensee, P. F. Jaeger, S. A. A. Kohl, J. Petersen, K. H. Maier-Hein, NnU-net: A self-configuring method for deep learning-based biomedical image segmentation. Nat. Methods **18**, 203–211 (2021). 33288961 10.1038/s41592-020-01008-z

[44] L. Wang, R.-C. Li, W.-W. Lin, Multiview orthonormalized partial least squares: Regularizations and deep extensions. IEEE Trans. Neural Netw. Learn. Syst. **34**, 4371–4385 (2021). 10.1109/TNNLS.2021.3116784 34637382

[45] J. Hu, L. Shen, G. Sun, “Squeeze-and-excitation networks” in Proceedings of the IEEE Conference on Computer Vision and Pattern Recognition (2018), pp 7132–7141.

[46] Y. Wang, K. Chen, Y. Zhang, H. Wang, Medtransformer: Accurate ad diagnosis for 3d mri images through 2d vision transformers. arXiv [Preprint] (2024) (Accessed 12 January 2024).

[47] R. Devi, V. Kumar, P. Sivakumar, Efficientnetv2 model for plant disease classification and pest recognition. Comput. Syst. Sci. Eng. **45**, 2249–2263 (2023).

[48] L. Yan , PSP net-based automatic segmentation network model for prostate magnetic resonance imaging. Comput. Methods Programs Biomed. **207**, 106211 (2021). 34134076 10.1016/j.cmpb.2021.106211

[49] E. Calabrese , Data from “The University of California San Francisco Preoperative Diffuse Glioma MRI (UCSF-PDGM)”. The Cancer Imaging Archive. 10.7937/tcia.bdgf-8v37. Deposited 30 November 2022.

[50] S. Bakas , Data from “Multi-parametric magnetic resonance imaging (mpMRI) scans for de novo Glioblastoma (GBM) patients from the University of Pennsylvania Health System (UPENN-GBM)”. The Cancer Imaging Archive. 10.7937/TCIA.709X-DN49. Deposited 21 June 2022.

[51] B. Erickson , Data from “LGG-1p19qDeletion”. The Cancer Imaging Archive. 10.7937/K9/TCIA.2017.DWEHTZ9V. Deposited 30 June 2017.

[52] U. Baid , Data from “Multimodal Brain Tumor Segmentation Challenge 2020”. Kaggle. https://www.kaggle.com/datasets/awsaf49/brats20-dataset-training-validation. Deposited 5 May 2020.

[53] CPTAC-GBM: National Cancer Institute Clinical Proteomic Tumor Analysis Consortium (CPTAC), Data from “The Clinical Proteomic Tumor Analysis Consortium Glioblastoma Multiforme Collection (CPTAC-GBM)”. The Cancer Imaging Archive. 10.7937/K9/TCIA.2018.3RJE41Q1. Deposited 10 May 2023.

[54] N. Shah , Data from “Ivy Glioblastoma Atlas Project (IvyGAP)”. The Cancer Imaging Archive. 10.7937/K9/TCIA.2016.XLwaN6nL. Deposited 30 December 2016.

[55] L. Scarpace , Data from “REMBRANDT”. The Cancer Imaging Archive. 10.7937/K9/TCIA.2015.588OZUZB. Deposited 12 September 2014.

[56] P. Juvekar , Data from “The Brain Resection Multimodal Imaging Database (ReMIND)”. The Cancer Imaging Archive. 10.7937/3RAG-D070. Deposited 26 September 2023.

[57] L. Scarpace , Data from “The Cancer Genome Atlas Glioblastoma Multiforme Collection (TCGA-GBM)”. The Cancer Imaging Archive. 10.7937/K9/TCIA.2016.RNYFUYE9. Deposited 7 August 2023.

[58] N. Pedano , Data from “The Cancer Genome Atlas Low Grade Glioma Collection (TCGA-LGG)”. The Cancer Imaging Archive. 10.7937/K9/TCIA.2016.L4LTD3TK. Deposited 29 May 2020.

[59] S. R. Van der Voort , Data from “The Erasmus Glioma Database (EGD)”. BMIAXNAT. https://xnat.health-ri.nl/REST/projects/egd. Deposited 2 June 2021. 10.1016/j.dib.2021.107191 PMC8203723 34159239

[60] Y. Suter , Data from “The LUMIERE Dataset: Longitudinal Glioblastoma MRI with Expert RANO Evaluation”. Figshare. 10.6084/m9.figshare.c.5904905.v1. Deposited 13 December 2022. PMC9755255 36522344

[61] H. Wang, OMT-APC. Github. 10.6084/m9.figshare.c.5904905.v1. Deposited 21 May 2025.

